# Singleton-Merten Syndrome: a rare autoimmune disorder caused by a specific IFIH1 mutation

**DOI:** 10.1186/2194-7791-2-S1-A12

**Published:** 2015-07-01

**Authors:** I Buers, Y Nitschke, RC Hennekam, M MacDougall, C Lu, O Mamaeva, GI Rice, H Erlandsen, HG Kehl, H Thiele, P Nürnberg, W Höhne, YJ Crow, A Feigenbaum, F Rutsch

**Affiliations:** Department of General Pediatrics, Muenster University Children's Hospital, Muenster, Germany; Department of Pediatrics, Academic Medical Center, University of Amsterdam, Amsterdam, the Netherlands; Institute of Oral Health Research, School of Dentistry, University of Alabama at Birmingham, AL USA; Manchester Academic Health Science Centre, Genetic Medicine, University of Manchester, Manchester, UK; Department of Pediatric Cardiology, Muenster University Children's Hospital, Muenster, Germany; Cologne Center for Genomics, University of Cologne, Cologne, Germany; Division of Clinical and Metabolic Genetics, Hospital for Sick Children, University of San Diego, San Diego, CA USA

## Meeting abstract

Singleton-Merten Syndrome (SMS) is a rare autosomal-dominant disorder characterized by a variety of symptoms including extreme calcifications of the ascending aorta and cardiac valves, dental anomalies (early onset periodontitis, root resorption), psoriasis and widened medullary cavities of the phalanges with focal osteoporosis. Employing whole exome sequencing of five SMS individuals of three families we identified the missense mutation, c.2465G>A (p.Arg822Gln) in the *interferon induced with helicase C domain 1* (*IFIH1*) gene. Additional Sanger sequencing confirmed co-segregation of the mutation with the disorder. *IFIH1*, encoding melanoma differentiation-associated protein 5 (MDA5), is a member of the RIG-I-like receptor family and functions as a cytoplasmic pattern recognition receptor recognizing viral double stranded RNA (dsRNA). Immunohistochemistry demonstrated the localization of MDA5 in all affected target tissues including heart, skin and cartilage. Functional analysis revealed that the *IFIH1* c.2465G>A mutation enhanced MDA5 function in interferon beta induction after polyinosinic:polycytidylic acid (poly (I:C)) stimulation in vitro. This indicates that SMS-MDA5 is hyperactive to non-self dsRNA. According to additional in vitro studies, interferon signature genes including SIGLEC1 were upregulated in blood and dental cells derived from SMS individuals. Taken together, our data demonstrate that the MDA5 gain-of-function mutation p.Arg822Gln causes the autosomal dominant disorder SMS through dysregulation of the human innate immune response.

